# *APC* Promoter Methylation in Gastrointestinal Cancer

**DOI:** 10.3389/fonc.2021.653222

**Published:** 2021-04-23

**Authors:** Lila Zhu, Xinyu Li, Ying Yuan, Caixia Dong, Mengyuan Yang

**Affiliations:** ^1^ Department of Medical Oncology, Key Laboratory of Cancer Prevention and Intervention, Ministry of Education, The Second Affiliated Hospital, Zhejiang University School of Medicine, Hangzhou, China; ^2^ Cancer Institute, Key Laboratory of Cancer Prevention and Intervention, Ministry of Education, The Second Affiliated Hospital, Zhejiang University School of Medicine, Hangzhou, China

**Keywords:** adenomatous polyposis coli, promoter, methylation, gastrointestinal cancer, CpG island

## Abstract

The adenomatous polyposis coli (*APC*) gene, known as tumor suppressor gene, has the two promoters 1A and 1B. Researches on *APC* have usually focused on its loss-of-function variants causing familial adenomatous polyposis. Hypermethylation, however, which is one of the key epigenetic alterations of the *APC* CpG sequence, is also associated with carcinogenesis in various cancers. Accumulating studies have successively explored the role of *APC* hypermethylation in gastrointestinal (GI) tumors, such as in esophageal, colorectal, gastric, pancreatic, and hepatic cancer. In sporadic colorectal cancer, the hypermethylation of CpG island in *APC* is even considered as one of the primary causative factors. In this review, we systematically summarized the distribution of *APC* gene methylation in various GI tumors, and attempted to provide an improved general understanding of DNA methylation in GI tumors. In addition, we included a robust overview of demethylating agents available for both basic and clinical researches. Finally, we elaborated our findings and perspectives on the overall situation of *APC* gene methylation in GI tumors, aiming to explore the potential research directions and clinical values.

## Introduction

The adenomatous polyposis coli (*APC*) gene is a tumor suppressor gene located in the human chromosome region 5q21–22 (1, 2). As a housekeeping gene, it consists of 15 exons and encodes a 300 kDa protein composed of 2,843 amino acids, which plays a vital role in cellular proliferation, migration, DNA repair, and chromosomal segregation (3). It was initially identified as the pathogenic gene in familial adenomatous polyposis (FAP), an autosomal dominant hereditary disease with numerous adenomatous polyps all over the colorectum (4, 5). Subsequently, it was shown to play an important role in other tumors, such as gastric and pancreatic cancers (6–9). One of the best-known mechanisms of *APC* is the regulation of the Wnt/*β*-catenin signaling pathway (10). Without a Wnt signal, *β*-catenin in the cell is firstly phosphorylated by the destruction complex composed by APC, glycogen synthase kinase 3*β* (GSK3*β*), Axin and casein kinase 1 (CK1), then ubiquitinated by *β*-TrCP200, and finally targeted for proteosomal degradation. In this process, the T cell factor/lymphoid enhancer factor (TCF/LEF) in the nucleus bands with the Groucho co-repressor protein to repress target genes. When the Wnt protein binds with the Frizzled (Fzd) receptor and low-density lipoprotein receptor related protein (LRP) co-receptor, they form a complex to activate the Dishevelled (Dsh) protein. Dsh and Axin bind together to help the formation of Wnt–Frz–LRP5/6 complex, which leads to a disassembled destruction complex, resulting in the stabilization and accumulation of *β*-catenin. This translocates into the nucleus and binds to a member of the TCF/LEF transcription factor family, activating the expression of many genes, such as the S-phase regulators c-myc and cyclin D1 to promote proliferation (11).

**Figure 1:**
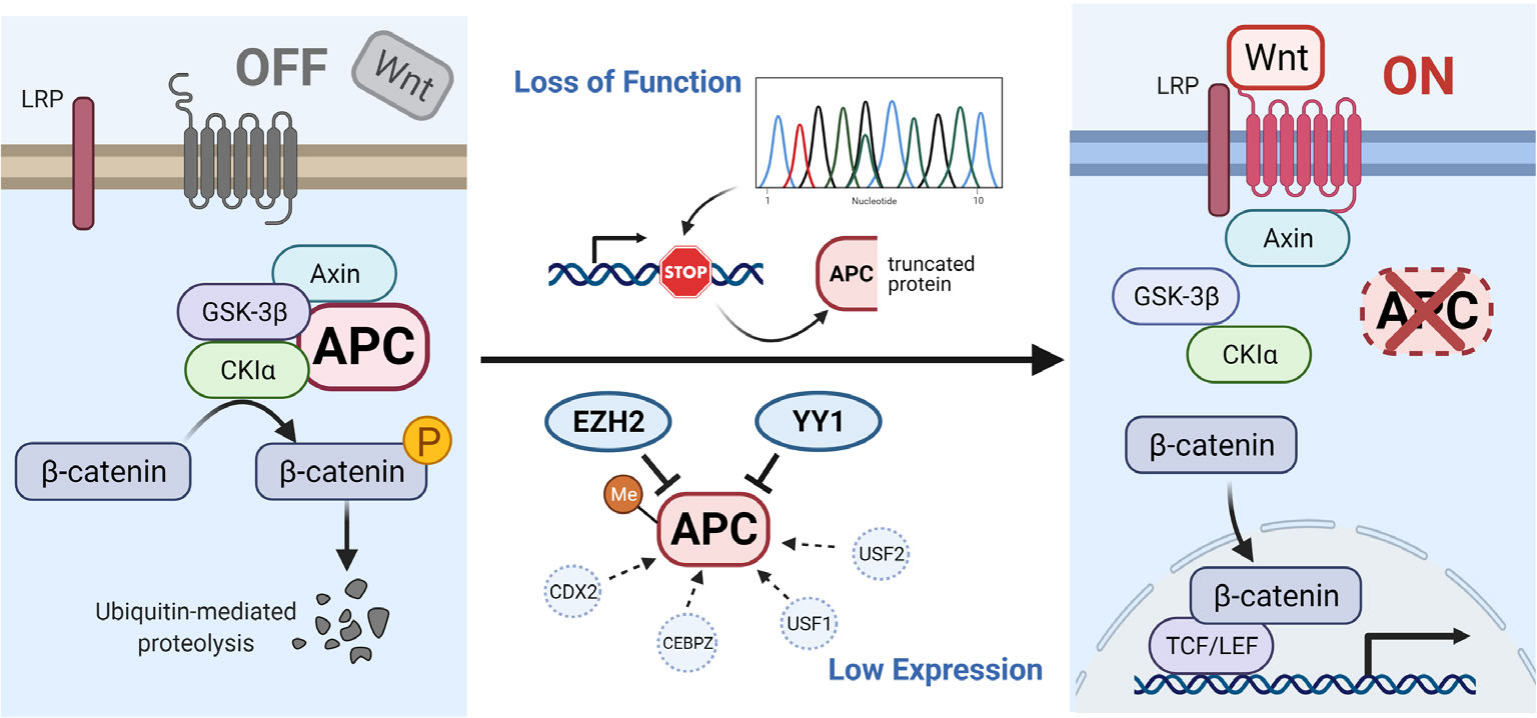
Illustration of APC gene-related pathways and interactive genes. In the absence of a Wnt signal (Wnt/*β*-catenin signaling pathway is inactive state, see left), the phosphorylated *β*-catenin can be formed by the destruction complex and degraded by ubiquitin-mediated proteolysis. Protein truncation generated by mutation in the APC gene or decreased level of APC protein influenced by other factors (such as hypermethylated APC promoter, improved expression of EZH2 and YY1, or suppressed expression of CDX2, CEBPZ, and USF1/2) can activate the canonical pathway (Wnt signaling active, see right). When the destruction complex disassembles, the *β*-catenin fails to be degraded and accumulates in the cytoplasm, translocates into the nucleus, and binds to TCF/LEF transcription factor family, causing the abnormal expression of downstream genes. The illustration was created with BioRender.com.

Mutations in *APC*, such as frameshift mutations, nonsense mutations or splice variants can induce premature stop codons and lead to the production of truncated *APC* proteins (12). In addition, certain other mechanisms can reduce the expression of *APC*, such as the hypermethylation of *APC* promoters (13–18), high expression of *EZH2* (19, 20) and *YY1* (21), or low expression of *CDX2* (22, 23), *CEBPZ* (24) and *USF*1/2 (25). All of these effects can lower the levels of destruction complex, causing the high *β*-catenin content to translocate into the nucleus and cause tumorigenesis (shown in **Figure 1**).

**Figure 2:**
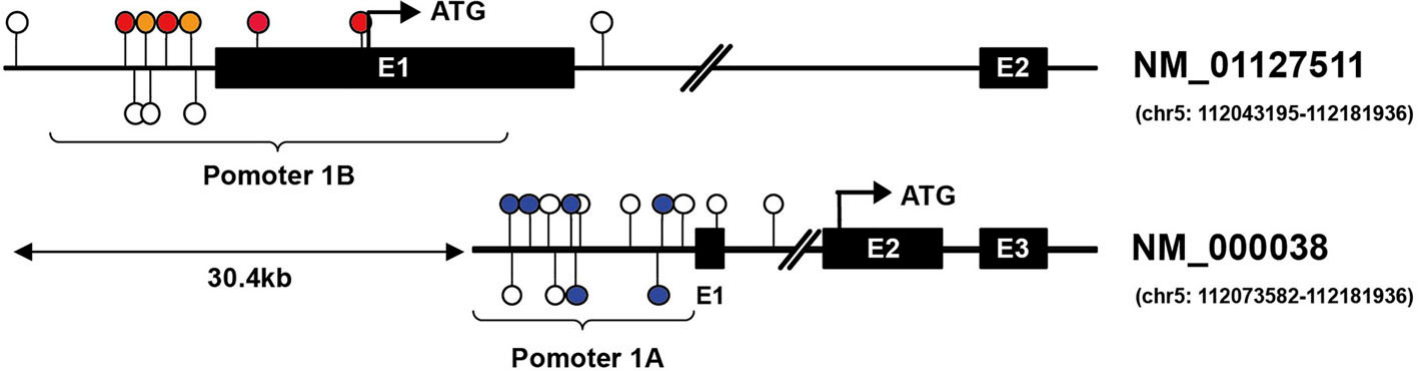
Illustration of CG locus in APC promoters in gastric and hepatic cancers. Promoter 1A (chr5: 112072720–112073585, GRCh37/hg19) is located at approximately 30.4 kb in the downstream region of promoter 1B (chr5: 112043008–112043594, GRCh37/hg19) of the APC gene. The colored lollipops denote statistically significant hypermethylated CG loci associated with prognosis, while the blue color indicates good prognosis, and the orange color means poor prognosis in gastric cancer; the red indicates poor prognosis in hepatic cancer [the colored lollipops referring to CG locus from left to right are cg04011030, cg18315896, cg08934600, cg18536802, cg08512345, cg25922032 (promoter 1B); cg00577935, cg14511739, cg14479889, cg16970232, cg23938220, cg02511809 (promoter 1A)]. Illustrator of biological sequences (IBS) was used to create the illustration.

In this review, we will focus on the impacts of hypermethylation of *APC* promoters on the development of gastrointestinal cancers. Two promoters have been identified in *APC*, termed as promoter 1A and promoter 1B. The latter is the most distal promoter of the *APC* gene [(GRCh37/Hg19) 112,043,008–112,043,594, 586 bp]. Promoter 1A is located at approximately 30.4 kb in the downstream region [(GRCh37/Hg19) 112,072,720–112,073,585, 865 bp], as shown as **Figure 2** (26, 27). Through alternative splicing, promoter 1A produces transcript 1A, and promoter 1B produces three transcripts, namely 1B1, 1B2, and 1B3 (28). Both promoters generate transcripts in all organs in the digestive tract, while the expression level of each transcript varies between body organs, thereby 1A and 1B have different importance (28). For example, compared with 1A, transcripts from *APC* 1B are predominant in normal colonic mucosae but not in the cerebrum (29–31).

**Figure 3:**
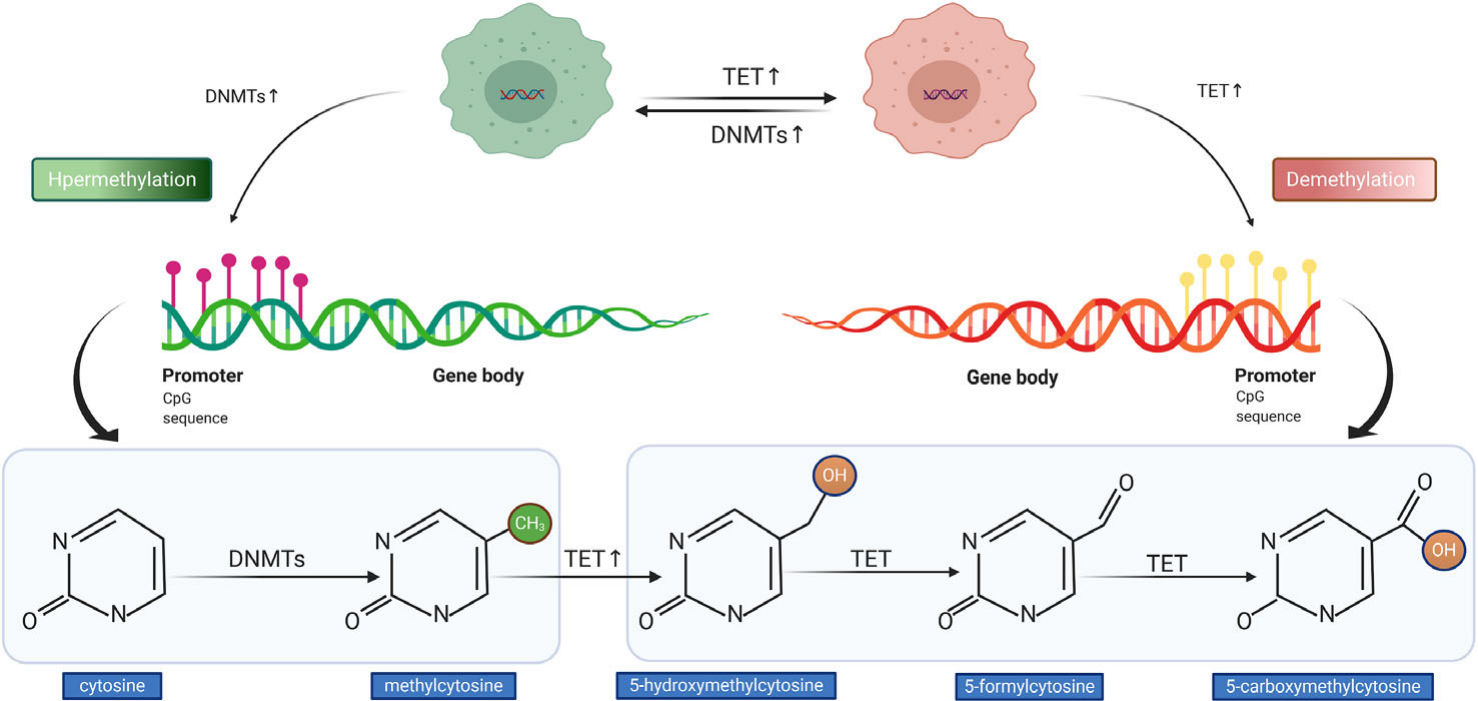
Illustration of DNA hypermethylation and demethylation patterns. The DNA methyltransferase (DNMT) mediates the hypermethylation of DNA promoter (left). The ten-eleven translocation (TET) enzyme conducts the demethylation of DNA promoter (right).

The process of DNA methylation is a common but a crucial event of epigenetic regulation, which frequently occurs in the CG-rich DNA sequence, the CpG island (32). Saxonov et al. determined that promoters of 72% of DNA were rich in CpG dinucleotides (33). CpG islands comprise organized CpG clusters with more than 50% content of guanine and cytosine (G + C) (34). In promoters, CpG islands generally remain in hypomethylated or unmethylated status to ensure that the gene expression machinery can normally access the promoters of target genes. As outlined in **Figure 3**, the balance between methylation and demethylation is maintained by enzymes with opposite functions, with the DNA methyltransferase (DNMT) and ten-eleven translocation (TET) enzymes playing major roles (35). The three described members of DNMT are DNMT1, DNMT3a, and DNMT3b, which could transform cytosine to 5-methylcytosine through promoting the covalent transfer of methyl groups to cytosine in the DNA sequence (36). Altered DNMT level or function could impact the DNA methylation status, which was observed in tumors with *APC* hypermethylation (37). On the contrary, TET enzyme recruitment boosts DNA demethylation, which accomplishes the demethylation role by oxidizing 5-methylcytosine (5mC) to 5-hydroxymethylcytosine and (5hmC) and 5-formylcytosine (5fC)/5-carboxycytosine (5caC) (35, 38). The measurement of methylation status can be performed by diverse techniques. The most widely used method in the studies cited in our review for detecting DNA methylation was methylation-specific PCR (MSP) followed by MethyLight assay, in addition to other methods such as methylation-sensitive single-strand conformation analysis, Illumina Infinium Human Methylation 450 BeadChip array, methylation specific-melting curve analysis (MS-MCA), and so on. The hypermethylation of *APC* promoter was found to be prevalent in most gastrointestinal (GI) cancers; it was detected in tumor tissues and even the corresponding serum of cancer patients, whereas it was generally less ubiquitous or absent in non-tumor tissues (39). The relationship between cancer and hypermethylation in *APC* CpG islands, however, has been shown to differ from cancer to cancer. In some GI cancers, *APC* hypermethylation could even be a potential indicator of early diagnosis or prognostic prediction (39, 40), while in others, it might be relative to tumorigenesis.

Although the studies dedicated to the role of hypermethylation of *APC* gene in digestive cancer have been thorough, a comprehensive knowledge of the mechanism is still not available. In this review, we attempt to expound an outline on the distribution of *APC* hypermethylation in GI cancers and promising demethylating agents that have been experimentally and clinically verified. We also discuss the underlying tumorigenesis mechanism and clinical value of *APC* hypermethylation.

## *APC* Methylation in Different Gastrointestinal Cancers

Most gastrointestinal tumors were shown to feature *APC* methylation, while data for duodenal cancer and cholecystic cancer are rare. Therefore, we focus on the introduction and analysis of the relationship between *APC* methylation and tumors of the digestive organs, except the duodenum and the cholecyst.

### Esophageal Cancer

There are two major subtypes of esophageal cancer: adenocarcinoma and squamous cell carcinoma. The pathogenic mechanism of *APC* reported for other tumors was mainly through truncated proteins caused by *APC* mutations, but these causative mutations reported for esophageal cancer have been rare (41). Surprisingly, hypermethylation in the *APC* promoter was detected both in tissue from esophagus adenocarcinoma and squamous cell carcinoma without any radiotherapy or chemotherapy, which occurred in esophageal tissues in 68–92% of patients with esophageal adenocarcinoma, in 44–50% of patients with esophageal squamous cell carcinoma, but not in matching normal esophageal tissues (39, 42–44), as shown specifically in **Table 1**.

**Table 1:** Methylation methods and methylation rate in each GI cancer.

Year	Sample size	Methylated rate	Detection method	*APC* promoter	Prognostic prediction	Nation	Reference
**Esophagus cancer**							
2000	84	76%	MSP	1A		USA	(39)
2006	27	92%	MS-SSCA; MS-DBA	1A		Switzerland	(42)
2004	50	78%	MSP	1A		Germany	(43)
2009	45	44.4%	MSP	1A		Iran	(44)
2009	59	45.8%	MSP	1A	wp	Germany	(45)
**Gastric cancer**							
2015	73	83.6%	MSP	1A		Greece	(46)
2018	24	83.3%	MSP	1A		China	(47)
2009	NA	NA	MSP	1A		Japan	(30)
2011	92	60%	MSP	1A		China	(48)
2016	40	82.5%	MSP	1A		Japan	(31, 49)
**Colorectal cancer**							
2013	52^s^/49^v^	53.8%^s^, 44.9%^v^	MSP	1A		Sweden & Vietnam	(50, 51)
1997	NA	NA	BSP	1A		Finland	(52)
2004	118	28%	MSP	1A		Canada & USA	(18)
2008	31	45%	MSP	1A		UK	(53)
2018	36^nsm^/47^fsm^/13^asm^	19%^nsm^, 26%^fsm^, 62%^asm^	IIHBM	1A		Germany	(54)
2012	112	11.9%	MSP	1A		Iran	(55)
2000	108	18%	MSP	1A		Spain and USA	(51)
2016	155	42%	MSP	1A	wp	Greece	(56)
2013	111	27%	Pyrosequencing	1A	wp	Sweden	(57)
**Liver cancer**							
2010	30	81.25%	IGMA	1A	wp	France	(58)
2014	57	64.91%	methylight	–	wp	China	(59)
2003	60	81.7%	MSP	1A		Korea	(60)
2015	221	78%	IIHBM	–		Italy & Spain	(61)
2011	32	84%	BSP	1A		China & USA	(62)
2013	23	100%^ss^	MSP	1A		Japan	(63)
**Pancreatic cancer**							
2020	NA	NA	IMBA	1A		Israel	(37)
2016	85	71%	MS-MCA	1A		Spain	(64)
2006	58	58.6%	MSP	1A		Japan	(65)

wp, worse prognosis; MSP, methylation-specific PCR; MS-SSCA, Methylation-sensitive single-strand conformation analysis; MS-DBA, Methylation-sensitive dot-blot assay; S, Swedish patients; V, Vietnamese patients; nsm, never smoking patients; fsm, former smoking patients; asm, active smoking patients; IIHBM, Illumina Infinium HumanMethylation450 BeadChip microarrays; NA, not available; BSP, Bisulfite sequencing PCR; IGMA, Illumina GoldenGate methylation assay; SS, samples from serum; IMBA, Infinium MethylationEPIC BeadChip Array; MS-MCA, methylation specific-melting curve analysis.

The hypermethylation of *APC* promoter was also observed in the plasma of patients suggested as hypermethylation positive for tumor tissue (39, 41, 45). Researchers found that the detection rate of *APC* methylation was higher in the blood of patients who had undergone surgery but featured residual tumor than that of patients with radical resection (45). Moreover, the hypermethylation level in the plasma increased with cancer stage (39, 41, 45). For patients with recurrent lesions, it was found that whether they had been plasma DNA-methylated or not, methylated *APC* could be detected in their plasma. It was further proposed that a high level of circulating methylated *APC* DNA and presence of tumor tissues was statistically significantly associated with reduced patient survival (39, 44).

According to these results, it is not difficult to conclude that the appearance of plasma DNA methylation may point to a more advanced tumor stage. Hypermethylated *APC* DNA can thus be a candidate biomarker to predict the tumor stage and provide prognosis. In addition, the detection of methylated *APC* in peripheral blood after surgery may provide crucial information about obvious residual tumors, potentially acting as a molecular “R0” marker (45).

### Gastric Cancer

Gastric cancer (GC) is one of the cancers with the highest mortality worldwide (66). Compared with patients without GC, the incidence of *APC* hypermethylation was reported as higher in the tissues and blood of patients with GC (67), which was 60 to 83.6% in these patients (46, 47, 49, 68) (listed in **Table 1**). The role of *APC* methylation in gastric cancer is more controversial than in other gastrointestinal tumors. In 2009, Hosoya et al. proposed that *APC* promoter 1A hypermethylation acted as a passenger in human gastric carcinogenesis (30). In contrast, the work of Balgkouranidou et al. showed that patients with *APC* methylation had lower survival, and the incidence of death was significantly higher in patients with methylated than those with unmethylated *APC* promoter status (46). Nevertheless, both studies indicated that the promoter 1A of *APC* gene was frequently methylated, while promoter 1B was unmethylated (30). In a different study, the promoter 1B of patients with primary GC was also completely in unmethylated status, while the underlying mechanism remained unknown (49).

Infection by *Helicobacter pylori* (Hp) was known as a risk factor for the development of benign lesions into GC, while the underlying mechanism has not been elucidated. Furthermore, Hp infection was reported to induce *APC* methylation (48). In addition to Hp, the inducible role of Epstein–Barr virus (EBV) in *APC* methylation was also demonstrated (68, 69). Therefore, patients with Hp or EBV infection should consider corresponding actively anti-infective treatment and as soon as possible.

### Colorectal Cancer

It is widely established that hereditary colorectal cancer (CRC) and sporadic CRC are the two main subtypes of colorectal cancer. Germline *APC* mutations are typically described as the cause of FAP, which is one of the hereditary forms of CRC (70). Concerning the role of *APC* in sporadic CRC, with the gradual deepening of researches on colorectal cancer, various studies have shown that the aberrant methylation of *APC* gene has a non-negligible impact on the occurrence and development of sporadic CRC (53, 54). The *APC* promoter methylation rate in CRC mucosa was detected in the range of 11.9 to 62% in different populations (18, 50, 51, 54, 55) (listed in **Table 1**). It was reported that hypermethylation occurred in multistage CRC tumorigenesis (53). According to certain studies, the heaviest CpG methylation occurred in colorectal cancer tissue, while it was slighter in adenoma and absent in normal colonic mucosa (40, 52). *APC* methylation was also measured in CRC patients at early or advanced stages (56).

As mutation appeared to be more common in cancer tissue, subsequent studies also measured the hypermethylation rate in *APC* promoter with or without mutation and found that hypermethylation was present in 20 of 108 (18%) tumors lacking *APC* mutation (51). In samples with mutant *APC*, however, only 2% demonstrated hypermethylated status. The remarkable finding was that, in FAP patients with certain germline mutations, hypermethylation was not detected at all (51). Thus, mutations in *APC* may antagonize the methylation of the *APC* promoter.

In a substantial proportion of sporadic CRCs, hypermethylation in the *APC* promoter can cause transcriptional silencing (40, 53). As a result of inactivation of *APC* transcription, transcripts expressed from 1A are significantly decreased, therefore it is quite likely that the Wnt signaling pathway may be activated, further causing cancer development in cells with hypermethylated *APC*.

*APC* methylation was also related to worse prognoses (56, 57). It was demonstrated that, independently of tumor stage, the survival outcome in methylation-negative CRC patients was better than that in methylation-positive patients (56).

### Liver Cancer

Known as one of the leading causes of death from cancer, hepatocellular carcinoma (HCC) has aroused growing attention in recent years. The progression of HCC was frequently related to epigenetic alterations of genes including *APC* (58–60). The *APC* promoter was reported to be hypermethylated in about 64.91 to 84% liver cancer patients (listed in **Table 1**), which was significantly higher than the non-HCC group (58–62), and a strong association was detected between hypermethylation and the risk of suffering from HCC (71). A relevant study specifically examined the DNA methylation status of the sense and antisense strand and demonstrated the occurrence of aberrant methylation in both, while methylation in the sense strand had a significantly higher specificity of 79% to distinguish HCC tissues from non-HCC tissues. Moreover, aberrant *APC* methylation was also detected in the serum of HCC patients (63), and serum AFP level and *APC* methylation status were proposed to be two independent markers for HCC. Methylation of the sense strand of *APC* was detected in 40% of patients with negative serum AFP (62). Consequently, the combination of serum AFP level and *APC* methylation may be a reliable approach to screen for HCC.

There is a level of controversy in researches on the relationship between APC methylation and the prognosis of patients with HCC. Augusto Villanueva et al. proposed that, in samples from tumor tissue, *APC* methylation was an indicator of lower survival in HCC patients (61). However, other researches have suggested that a higher degree of CpG island methylation of *APC* promoter in tumor tissue was not a definitive marker of lower overall survival rate (58, 59). In vascular circulation samples, higher *APC* methylation was related to vascular invasion (63). Based on these varied results, more in-depth research will be needed to clarify the relationship between *APC* methylation and HCC.

### Pancreatic Cancer

One of the most aggressive cancers is pancreatic cancer (PC). Compared with other GI cancers, less attention has been paid on the correlation between *APC* promoter methylation and pancreatic cancer. A number of early studies found that the frequency of *APC* promoter CpG island hypermethylation was quite distinguished and was detected in 58.6 to 71% of patients with pancreatic cancer (64, 65) (shown in **Table 1**). Neuroendocrine pancreatic cancer, but not the major population of pancreatic cancer, also exhibits hypermethylation in *APC* promoter 1A. The overexpression of DNMT1 was detected in the tissue with hypermethylated *APC*, which might account for this phenomenon (37). Results of He et al. showed that DNMT was regulated by transcriptional factor GLI, and *APC* methylation in PC might be promoted by DNMT upregulation (72).

### Data From Public Databases

MethSurv is a web tool to perform multivariable survival analysis based on CpG methylation patterns and includes 7,358 methylomes from 25 different human cancers. It utilizes methylome data from Illumina Infinium HumanMethylation450 (HM450K). The microarray data is sourced from The Cancer Genome Atlas and GDAC Firehose, and the Cox proportional-hazards model is used to develop an interactive web interface for survival analysis. MethSurv enables survival analysis for a single CpG located in or around the proximity of a query gene. What is more, the clinical characteristics and optional browsing of top biomarkers for each cancer type are also available (73).

Upon analyzing the data downloaded from MethSurv, it is found that the level of methylation in *APC* promoters may be concerned with the prognosis and clinical stage of gastrointestinal cancers. In gastric cancer, for instance, the hypermethylation of CG locus in *APC* promoter 1A indicates good prognosis and has negative correlation with clinical stage, while the partial CG locus (red lollipops in **Figure 2**) in *APC* promoter 1B suggests that hypermethylation is closely associated with poor prognosis. Hypermethylation in some CG loci (orange lollipops in **Figure 2**) of *APC* promoter 1B in hepatic cancer prompts less median survival time, and the DNA methylation level in cg08512345 appears positively correlated with clinical stage. However, unlike in gastric cancer, most of the hypermethylated CG loci located in promoter 1A in pancreatic cancer demonstrate poor prognosis and are also positively correlated with clinical stage (except cg21634602), as summarized in **Table 2**. As opposed to gastric, hepatic, and pancreatic cancer, we find that, for esophageal cancer and colorectal cancer, whether in promoter 1A or 1B, the correlations between CG locus methylation level and prognosis or clinical stage are insignificant. Therefore, continued exploration is required to establish the exact role of CG locus hypermethylation in different cancers of the gastrointestinal tract.

**Table 2:** Hypermethylation of APC promoters associated with prognosis in pancreatic cancer.

CpG site	Position	*APC* promoter	High (n)	Low (n)	p value	Prognosis	HR	95%CI
cg15020645	112,073,769	1a	138	46	0.012	harmful	0.544	(0.331;0.895)
cg24332422	112,073,686	1a	138	46	0.024	harmful	0.580	(0.352;0.954)
cg12534150	112,073,613	1a	46	138	0.0025	harmful	0.489	(0.314;0.761)
cg21634602	112,073,570	1a	46	138	0.001	harmful	0.474	(0.310;0.725)
cg02511809	112,073,544	1a	92	92	0.013	harmful	0.596	(0.395;0.899)
cg23938220	112,073,538	1a	46	138	0.021	harmful	0.582	(0.372;0.908)
cg20311501	112,073,502	1a	138	46	0.018	harmful	0.573	(0.354;0.926)
cg03667968	112,073,438	1a	46	138	0.035	harmful	0.611	(0.392;0.951)
cg16970232	112,073,433	1a	138	46	0.023	harmful	0.582	(0.357;0.949)
cg14479889	112,073,426	1a	95	89	0.039	harmful	0.654	(0.436;0.980)
cg11613015	112,073,406	1a	66	118	0.0028	harmful	0.526	(0.348;0.797)
cg22035501	112,073,398	1a	71	113	0.019	harmful	0.608	(0.403;0.917)
cg14511739	112,073,373	1a	75	109	0.021	harmful	0.617	(0.411;0.925)
cg08571859	112,073,350	1a	138	46	0.073	harmful	0.664	(0.420;1.052)
cg00577935	112,073,348	1a	92	92	0.049	harmful	0.668	(0.446;0.998)
cg04226363	112,043,716	1b	46	138	0.098	protective	1.504	(0.910;2.487)
cg25922032	112,043,407	1b	92	92	0.094	harmful	0.711	(0.477;1.060
cg08512345	11,204,3275	1b	78	106	0.065	harmful	0.680	(0.454;1.019)
cg26660754	112,043,194	1b	46	138	0.21	protective	1.353	(0.827;2.212)
cg18536802	112,043,188	1b	92	92	0.41	protective	1.185	(0.794;1.767)
cg08934600	112,043,158	1b	138	46	0.34	protective	1.245	(0.799;1.938)
cg01528425	112043137	1b	87	97	0.52	protective	1.142	(0.765;1.704)
cg18315896	112043131	1b	46	138	0.33	harmful	0.797	(0.508;1.252)
cg16481008	112043117	1b	80	104	0.05	protective	1.502	(0.993;2.273)
cg11057897	112042966	1b	138	46	0.28	harmful	0.781	(0.494;1.235)

HR, hazard ratio; CI, confidence interval.

## Drug Resistance Related to DNA Methylation

Resistance to therapeutic drugs is a significant challenge affecting survival and prognosis for patients receiving medical treatment. Relevant studies have shown that, when a certain gene was abnormally methylated, the abnormal expression of that gene and resistance to anti-tumor drugs would develop (74–77). Shen et al. reported that decreased expression of the tumor suppressor gene *KANK1* could increase paclitaxel resistance in non-small cell lung cancer (75). In CRC, *NKX6.1* gene was demonstrated to participate in the regulation of chemotherapy sensitivity for its hypermethylation (78).

Limited research has focused on the relationship between drug-resistance and *APC* methylation. While studying the association between the methylation of multiple genes and sensitivity to pyrimidine synthesis inhibitors, such as cyclocytidine and AraC, researchers found that *APC* methylation was linked to cell sensitivity to this kind of drug (79). Some studies only exhibited a directed correlation between *APC* expression level and drug resistance (74, 80). The loss-of-function of *APC* was able to decrease sensitivity to doxorubicin (DOX) of triple negative breast cancer (TNBC) in the study of Stefanski et al. (80). Through cooperating with *MDM2* (murine double minute 2 gene), *APC* might be involved in drug resistance in ovarian cancer (74). It is well-established that *APC* is abnormally methylated in both breast cancer (81, 82) and ovarian cancer (83, 84), therefore it cannot be ruled out that *APC* might affect cancer cell responsiveness to anti-tumor drugs through gene silencing induced by promoter methylation. However, specific experiments are needed to prove this postulation.

## Potential Agents Exhibiting a Demethylating Effect

DNA hypermethylation may lead to gene silencing in the promoter region, while demethylation can reactivate gene expression (85–87). Relative agents used in modifying the hypermethylated status of different genes have emerged over the years, with the two most widely applied demethylating drugs 5-azacytidine (azacytidine) and 5-aza-2′-deoxycytidine (decitabine, DAC) (88–91). For example, in patients with myelodysplastic syndrome (MDS) and acute myeloid leukemia (AML), azacytidine and DAC were used to demethylate tumor suppressor genes like *CDH1* and *CDNNA1*, in order to achieve therapeutic effects (92–94).

Apart from MDS and AML, researches on demethylation agents also made some progress in certain solid tumors (95–98). On the one hand, ongoing clinical trials of these drugs to reverse the methylation status in genes have shown varying efficacy and promising results (93, 99). On the other hand, basic research also became intensive. In liver cancer, for example, arsenic trioxide (ATO), an ancient chemotherapy drug, could repress the NF-*κ*B pathway through miR-148a demethylation, and further reverse the resistance of tumor cell (97). Moreover, DAC was able to improve the sensitivity of gastric cancer to irinotecan cisplatin through the demethylation of promoter CpG islands of *RUNX3* and *PYCARD* (98).

In addition to common drugs like DAC, researches on further new agents gradually emerged (16, 100, 101), and some of them could even exhibit a demethylating effect on the *APC* gene. Selenite treatment was demonstrated to reduce levels of DNMT1 and reactivate methylation-silenced gene expression in prostate cancer cells (16). In another study, genistein was capable of competitively binding with DNMT1, which inhibited the interaction of DNMT1 and *APC* DNA to protect *APC* from methylation. By treating breast cancer cells presenting hypermethylated *APC* with genistein, the methylation-induced silencing in *APC* was reversed. Natural DNMT inhibitors, such as epigallocatechin-3-gallate (EGCG) and curcumin, also exerted inhibitory effects by competitively binding with DNMT1 (101, 102).

In pancreatic cancer, *APC* hypermethylation was inferred to result from DNMT upregulation (37, 72), thus agents targeting DNMT might be possible therapeutic drugs for PC. The expression of DNMT was not demonstrated in other GI cancers, therefore it remains uncertain whether the anti-DNMT drug effect potentially occurs in these cancers.

## Conclusions and Perspectives

In the relevant literature, *APC* hypermethylation was found tumor-specific in most gastrointestinal cancers, and was suggested as a potential alternative tumorigenesis mechanism for GI cancers. In some GI cancers, *APC* hypermethylation may serve as a candidate molecular marker for early disease screening, clinical stage, prognostic prediction and drug efficacy. Nowadays, anti-cancer treatment is gradually moving toward precise and individualized treatment, and APC methylation can be used as a novel target for treating malignant tumors. A rising number of agents are found to reverse the hypermethylated status of *APC*, which can be a promising anti-cancer method, while more research is necessary to establish such effective therapeutic alternatives. The future of CRISPR Cas9 and other gene editing methods to achieve demethylation can also have significant importance for cancer therapy.

## Author Contributions

LZ was mainly responsible for writing conception, literature searching, and drafting of the manuscript. XL participated in the collection of literatures and revision of article. YY was responsible for the revision of the manuscript. CD provided professional revision for the article. MY participated in the conception and revision of the article. All authors contributed to the article and approved the submitted version.

## Funding

This work was supported by the National Key R&D Program of China (2017YFC0908200), the National Natural Science Foundation of China (81872481), and Traditional Chinese Medicine (Integrated Chinese and Western Medicine) Key Discipline Construction Project of Zhejiang Province (2017-XK-A40).

## Conflict of Interest

The authors declare that the research was conducted in the absence of any commercial or financial relationships that could be construed as a potential conflict of interest.
